# Historical trends and future projections of cervical disc arthroplasty and removal cervical disc arthroplasty in the United States Medicare population

**DOI:** 10.1016/j.xnsj.2025.100774

**Published:** 2025-07-20

**Authors:** Tarun Mattikalli, Jeremy Steinberger, Konstantinos Margetis

**Affiliations:** Department of Neurosurgery, Icahn School of Medicine at Mount Sinai, 1 Gustave L. Levy Place, New York, NY 10029, United States

**Keywords:** Cervical arthroplasty, Cervical disc replacement, Medicare claims, Procedure volume, Forecast modeling, Time series analysis, CMS claims data

## Abstract

**Background:**

Cervical disc arthroplasty (CDA) has become an increasingly utilized alternative to anterior cervical discectomy and fusion (ACDF), offering potential benefits such as motion preservation and reduced incidence of adjacent segment disease. However, long-term utilization trends and future procedural burden remain unclear.

**Methods:**

Medicare fee-for-service (FFS) cervical disc arthroplasty (CDA) volumes were extracted from the Medicare Part B National Summary between 2009 and 2022, excluding 2020, and uplifted to account for Medicare Advantage enrollment. Statistical models developed included ordinary least squares (OLS), generalized linear models (GLM), and segmented regression to detect any inflection points in utilization. Autoregressive and alternative GLMs such as Poisson and negative binomial were used for validation. For each procedure type, the model that best aligned with observed trends and statistical fit (AIC, R^2^) was used to generate forecasts through 2035.

**Results:**

Primary CDA utilization demonstrated an inflection point in 2018, after which growth stabilized. Post 2018 OLS modeling estimated a 6.2% annual growth rate, reaching an estimated 9,422 procedures by 2035 (95% CI: 5,494–16,159). Removal CDA exhibited consistent exponential growth, with GLM estimated 22.9% annual increase and a projected volume of 1,773 procedures by 2035 (95% CI: 1,183–2,656).

**Conclusions:**

Primary CDA may be entering a mature adoption phase with modest projected growth, while removal CDA is expected to grow more substantially. These projections reflect trends in an older population, which may not generalize to younger individuals undergoing CDA. Our findings reinforce the need for continued surveillance, resource planning, and better understanding of long-term CDA complications in the increasingly aging population.

## Higlights

• Medicare data was used to project CDA trends through 2035.
• Segmented regression identified inflection point in primary CDA utilization.
• Primary CDA volume growth plateaued post 2018, and is estimated to reach about 9,400 by 2035.
• Removal CDA is projected to grow about 22.9% annually, reaching about 1,800 cases annually by 2035.
• Findings inform planning for CDA complications, removals, and aging populations.

## Introduction

Cervical disc arthroplasty (CDA) has gained traction over the past few decades as an alternative to anterior cervical discectomy and fusion (ACDF) for treating cervical disc disease in appropriately indicated patients [1]. While numerous studies have demonstrated comparable outcomes in both CDA and ACDF [[2], [3], [4], [5]], there is concern that fusion may increase likelihood of accelerated degeneration of adjacent levels of the spine [6,7]. CDA, on the other hand, preserves motion at the operative level, which may reduce the incidence of adjacent segment disease [8,9]. In some studies, CDA has been associated with lower reoperation rates and improved longer-term outcomes [[10], [11], [12]]. CDA is therefore increasingly adopted for both single-level and 2-level cervical disc disease, as well as more complex cases, such as multilevel disease, and patients with relative contraindications [13,14]. Despite these encouraging results, potential complications such as heterotopic ossification [15,16], device migration and subsidence [16,17], or osteolysis [18] can necessitate removal and/or revision of CDA.

As adoption, revision, and removal of CDA have matured, questions remain regarding its ideal indications, long-term utilization patterns, revision rates, and future procedural burden on the national healthcare system. While population-level projections have been published for certain orthopedic procedures, comparable national projections for CDA remain limited. Understanding utilization trends and forecasting future procedure volumes is critical for surgical workforce planning, implant design, payer policies, and operative resource allocations.

Therefore, our study aims to provide updated, long-term projections of primary and removal cervical disc arthroplasty procedures using national Medicare claims data. This dataset primarily reflects an older U.S. population, and therefore the trends described may not be generalizable to younger patients or those in other insurance groups. We applied established statistical modeling methodology to characterize historical trends and estimate future growth. While true revision CDA procedures could not be reliably modeled due to data suppression, this study provides a comprehensive national projection of national primary and removal CDA volumes.

## Methods

This study utilized annualized Fee-For-Service (FFS) Medicare procedure counts from the CMS Medicare Part B National Summary [19] between 2009 and 2022, collected in April 2025. As this study only utilized de-identified and publicly available data, institutional review board (IRB) approval was not required. Data from 2020 were excluded due to the well-documented reduction in elective surgical procedures during the COVID-19 pandemic [20], which led to a temporary but significant decline in cervical disc arthroplasty volumes across all payer groups [21]. Inclusion of this outlier year would introduce bias into trend modeling and long-term projections. Cervical disc arthroplasty (CDA) procedures were identified using Current Procedural Terminology (CPT) codes and categorized into primary (CPT 22856), removal (CPT 22864), and revision (CPT 22861) groups based on published literature [1,[22], [23], [24]] and the NLM database [25]. Annual procedure volume data was collected by type, and independent statistical models were developed for each category where sufficient data was available.

Procedure counts for revision cervical disc arthroplasty (CPT 22861) were consistently below CMS reporting threshold (fewer than 11 performed) and therefore suppressed for privacy. Due to the lack of reliable year-over-year data, revision CDA was excluded from the modeling and forecasting in this study. Analysis was performed for only primary cervical disc arthroplasty (CPT 22856) and removal cervical disc arthroplasty (CPT 22864).

As these procedure counts only included fee-for-service (FFS) patients and not Medicare Advantage (MA) patients, numbers were uplifted to extrapolate the total number of procedures performed yearly using a ratio of FFS to MA patients, provided by Kaiser Family Foundation [26]. The total number of FFS and uplifted primary cervical arthroplasty procedures and removal arthroplasty procedures are displayed in Table 1, Table 2

**Table 1:** Yearly primary and removal cervical disc arthroplasty procedure volumes, year-over-year change, and yearly percentage increase

CMS data						
Year	Primary CDA			Removal CDA		
	Volume	YOY change	% Change	Volume	YOY change	% Change
2009	121	-	-	5	-	-
2010	128	7	5.8%	5	0	0.0%
2011	167	39	30.5%	13	8	160.0%
2012	145	−22	−13.2%	5	−8	−61.5%
2013	218	73	50.3%	20	15	300.0%
2014	408	190	87.2%	13	−7	−35.0%
2015	617	209	51.2%	19	6	46.2%
2016	1033	416	67.4%	31	12	63.2%
2017	1525	492	47.6%	26	−5	−16.1%
2018	2091	566	37.1%	35	9	34.6%
2019	2260	169	8.1%	34	−1	−2.9%
2020	2029	−231	−10.2%	58	24	70.6%
2021	2194	165	8.1%	55	−3	−5.2%
2022	2226	32	1.5%	63	8	14.5%

**Table 2:** Yearly primary and removal cervical disc arthroplasty procedure volumes, year-over-year change, and yearly percentage increase, uplifted to account for Medicare advantage enrollment

Uplifted CMS data							
Year	% MA Patients	Primary CDA			Removal CDA		
		Volume	YOY change	% change	Volume	YOY change	% change
2009	24%	159	-	-	7	-	-
2010	25%	171	11	7.2%	7	0	1.3%
2011	26%	226	55	32.2%	18	11	163.5%
2012	27%	199	−27	−12.0%	7	−11	−61.0%
2013	29%	307	108	54.6%	28	21	311.3%
2014	31%	591	284	92.6%	19	−9	−33.1%
2015	32%	907	316	53.4%	28	9	48.3%
2016	33%	1542	634	69.9%	46	18	65.6%
2017	35%	2346	804	52.2%	40	−6	−13.5%
2018	37%	3319	973	41.5%	56	16	38.9%
2019	39%	3705	386	11.6%	56	0	0.3%
2020	42%	3498	−207	−5.6%	100	44	79.4%
2021	46%	4063	565	16.1%	102	2	1.9%
2022	48%	4281	218	5.4%	121	19	19.0%

The modeling framework was based on prior orthopedic procedure projection studies using ordinary least squares (OLS) and generalized linear modeling (GLM) techniques with a log link [27,28]. OLS (log-linear) was used for stable trends with normally distributed residuals after log transformation, while GLM (log-link) was selected for greater variability or consistent exponential growth behaviors.

Alternative fits were evaluated using general linear model methods such as Poisson and negative binomial regression and autoregressive integrated moving average (ARIMA) for validation. To detect any major shifts in utilization patterns over time, a segmented linear regression model was applied to the log-transformed procedure volumes [29]. If a statistically significant inflection point was identified, revised OLS and GLM models using only postinflection data were considered. The final model for each group was selected based on statistical fit using Akaike Information Criteria, interpretability, and alignment with observed utilization trends. The model that best satisfied these criteria after validation was then used to generate procedure volume projections through 2035.

All statistical analysis was performed using the R programming environment version 4.4.3 (R Core Team 2025) R: A Language and Environment for Statistical Computing. R Foundation for Statistical Computing, Vienna, Austria, https://www.R-project.org.

## Results

### Primary cervical disc arthroplasty

Segmented regression identified a statistically significant inflection point in primary CDA usage in 2018 (estimated break point: *t*=9.67, SE=1.06), marking a shift from rapid growth to a potentially plateauing trend. Before the inflection point (2009-2017), procedure volumes exhibited a more rapid exponential growth, with a log-linear slope of 0.3667, corresponding to an estimated annual growth rate of 44.3%. After the inflection point, the growth rate declined to a slope of 0.0479, or an estimated annual growth rate of 4.91%.

**Fig. 1:**
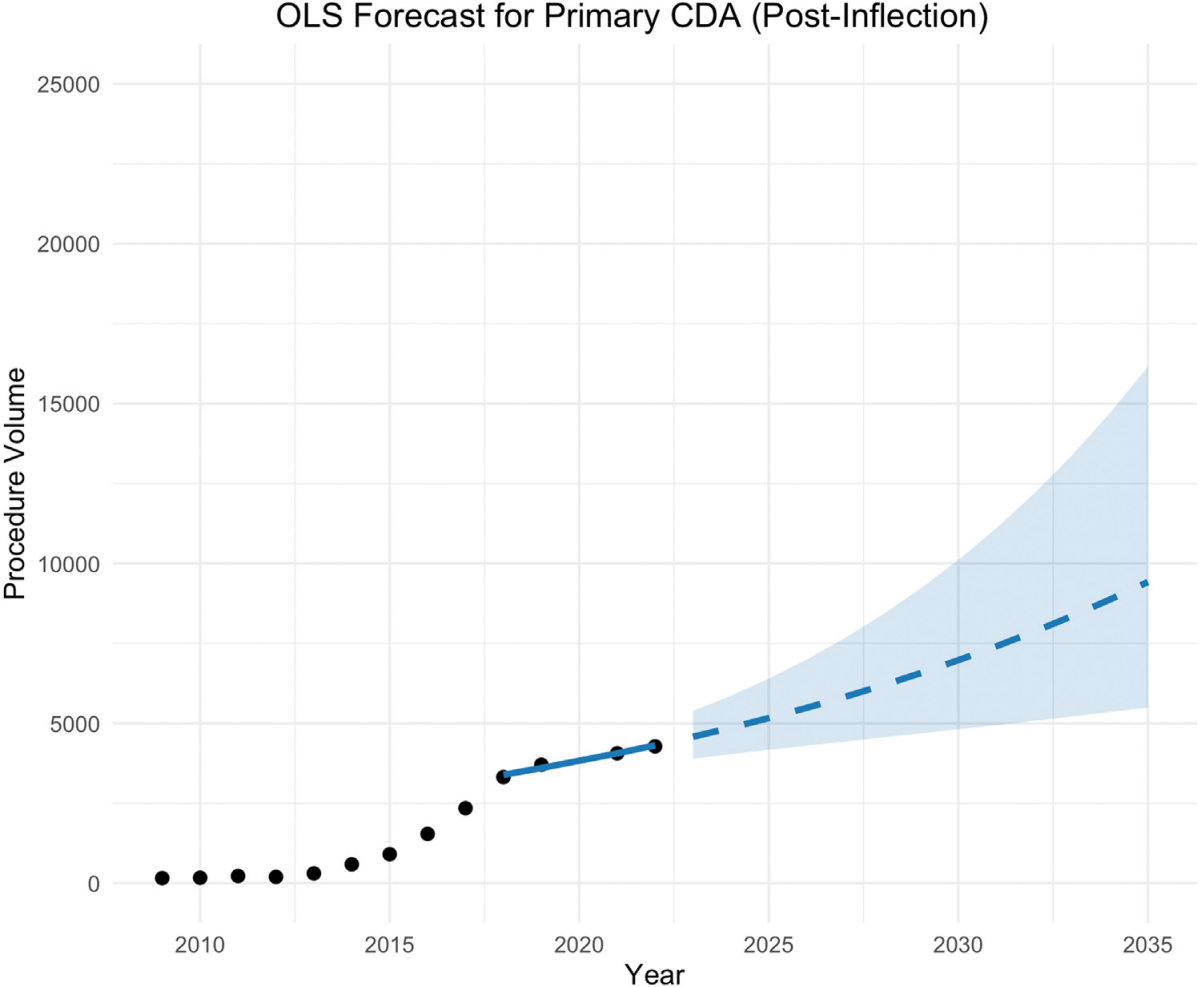
Primary CDAs projected between 2023 and 2035 using OLS log-linear model. Black dots represent uplifted CMS data from 2009 to 2022 (excluding 2020). Blue line represents model fit from 2018 to 2022 (post inflection), and blue dotted line represents forecast. Blue shaded area indicates 95% confidence intervals.

Due to this observed stabilization after 2018, an ordinary least squares (OLS) model was fit to log-transformed data from 2018 to 2022 only. The model predicted an estimated annual growth rate postinflection was 6.2% (p=.018), with the model fit reaching R^2^=0.947, AIC=−14.7, and SE=0.81 (Table 3). Forecasting from 2023 to 2035 primary CDA procedure volume is expected to reach 9,422 by 2035 (95% CI=5,494-16,159). Full yearly projections and confidence intervals are presented in Table 4 and visualized in Fig. 1.

**Table 3:** Regression estimates and growth rates with 95% confidence intervals formulated using normal approximations

Model used	Procedure Type	Intercept	Trend estimate	Standard error	Projected annual growth (%) - confidence intervals obtained by normal approximation		
	y	β_0_	β₁	S.E. of β₁	Average	95% CI, lower	95% CI, upper
OLS, log-linear	Primary CDA	8.13	0.601	0.0018	6.20%	4.50%	7.90%
GLM, log-link	Removal CDA	2.12	0.206	0.0014	22.90%	19.70%	26.20%

Model selection (OLS vs. GLM) was based on statistical fit and alignment with observed trends.

**Table 4:** Primary CDA and removal CDA projections with low and high 95% confidence intervals and cumulative projected growth

Forecasted year (2-year intervals)	Primary CDA (OLS)			Removal CDA (GLM)		
	Projection	95% CI	Cumulative growth (Base = 2022)	Projection	95% CI	Cumulative growth (Base = 2022)
2023	4,580	3,894–5,387	6.99%	149	135–165	22.98%
2025	5,165	4,169–6,400	20.66%	225	194–261	85.71%
2027	5,825	4,427–6,999	36.07%	340	280–414	180.63%
2029	6,569	4,683–9,215	53.45%	514	401–658	324.25%
2031	7,408	4,944–11,101	73.05%	777	576–1,047	541.33%
2033	8,355	5,214–13,388	95.18%	1173	825–1,668	868.19%
2035	9,422	5,494–16,159	120.10%	1773	1,183–2,656	1,363.43%

### Removal of cervical disc arthroplasty

**Fig. 2:**
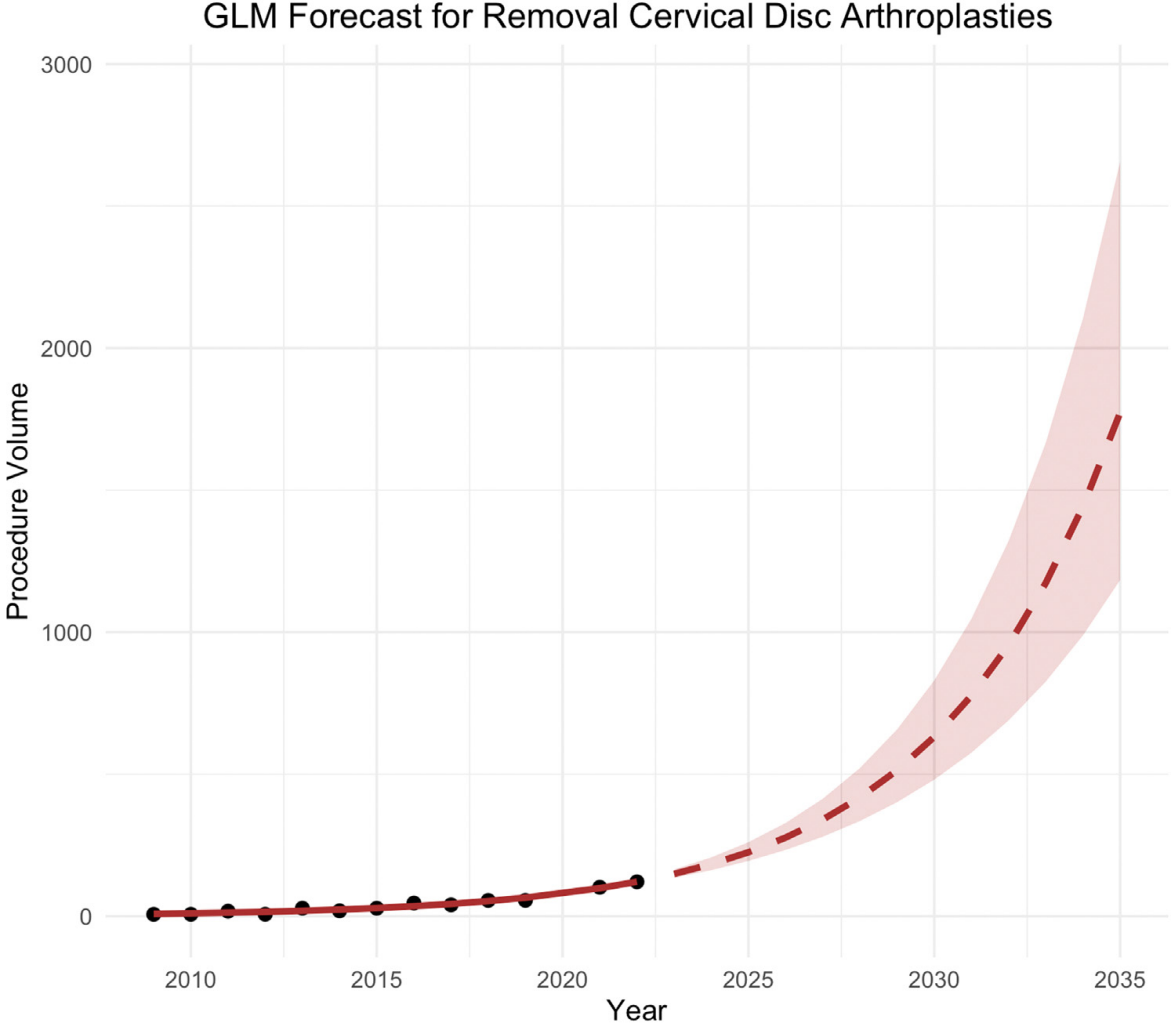
Removal CDAs projected between 2023 and 2035 using GLM log-link model. Black dots represent uplifted CMS data from 2009 to 2022 (excluding 2020). Red line represents model fit from 2009 to 2022, red dotted line represents forecast. Red shaded area indicates 95% confidence intervals.

Removal of cervical disc arthroplasty demonstrated a more consistent growth pattern throughout the study period. A generalized linear model (GLM) with gaussian distribution and log-link best modeled procedure volumes from 2009 to 2022. This model achieved an AIC of 89.3, with statistically significant intercept and slope coefficients (p<.001). An annual log-linear slope of 0.2064 was estimated, corresponding to an estimated annual growth rate of 22.9% (Table 3) from 2023 to 2035. By 2035, removal CDA volume is estimated to reach 1,773 (95% CI = 1,183-2,656). Yearly forecasts between 2023 and 2035 are presented in Table 4 and Fig. 2.

## Discussion

This study provides a detailed analysis and forecast of cervical disc arthroplasties (CDA) utilization within the Medicare population, highlighting distinct growth patterns for primary and removal CDA procedures. Using Medicare claims data from 2009 – 2022 and statistical modeling, we identified clear trends in procedure adoption and device removal among older adult beneficiaries, projecting these trends forward through 2035.

It is critical to contextualize our projections within the broader U.S. cervical disc arthroplasty landscape. Medicare beneficiaries, the population studied here, account for a relatively small fraction of total CDA recipients. In a large nationally representative sample claims database (PearlDiver M151Ortho, 2010–2021), only 4.8% of CDA procedures were performed in Medicare-insured patients, compared to 86.5% covered by commercial insurances and 5.0% by Medicaid [21]. This is consistent with the lower proportion of older adults undergoing CDA observed across other datasets [30,31]. Given this payer distribution, our findings, while internally valid for Medicare patients, may not fully capture utilization trends or revisions risks in the younger, commercially insured population that may constitute the majority of CDA cases.

We observed a significant inflection in primary CDA usage around 2018, transitioning from a rapid growth period to a markedly slower, potentially plateauing growth trend after. The early phase of CDA growth, with an annual increase of 44.4%, aligns closely with previously published trends showing rapid adoption of CDA following newer-generation implants [23]. Existing studies based on large administrative and clinical databases have shown consistent early enthusiasm for CDA driven by perceived advantaged over traditional ACDF, such as preserved segmental motion, reduced risk of adjacent segment degeneration, and potentially quicker recovery [[32], [33], [34]]. Post inflection estimate of annual growth of 6.2% after 2018 suggests that CDA has entered a more mature adoption phase with plateaued growth. Other studies have found a similar pattern in CDA utilization [33,35], and have attributed this to possible regional, racial, and sex disparities, as well as a more strict approach in selection of CDA over traditional ACDF [36]. Our projections indicate that primary CDA procedures may approach 9,422 annually by 2035, highlighting the need for optimal decision making (ACDF vs. CDA), training, and patient counseling as CDA adoption stabilizes.

In contrast to primary CDA, removal CDA procedures exhibited sustained exponential growth, with an estimated annual growth rate of 22.9%. This rapid increase may represent a natural outcome of the earlier rapid adoption phase, mirroring a similar lifecycle seen in other spinal implants, such as lumbar disc replacements and stabilization devices [[37], [38], [39]]. Furthermore, as CDA usage increased substantially over the previous decade, long-term complications have been increasingly reported and may require surgical removal [[40], [41], [42]], including radiculopathy, heterotopic ossification, and device migration [[15], [16], [17]]. Osteolysis may also necessitate removal of CDA [18,43,44], which has also been well documented as a frequent indication for revision in hip and knee arthroplasties [[45], [46], [47]].

Implant design characteristics can influence risk of complications leading to removal or revision. One study analyzing outcomes of the M6-C cervical disc replacement reported a 34% mid-term revision rate due to wear related osteolysis, with an overall osteolysis prevalence of 44% [48]. Similarly, the Mobi-C implant has been associated with instances of segmental kyphosis due to the device being locked in a flexed position, necessitating reoperation [49]. These findings underscore that implant-specific factors, such as material composition and biomechanical design, may contribute varying rates of complication. However, due to the absence of device-specific data in larger databases, further research utilizing registries or datasets with device identifiers may strengthen these associations.

Economic factors relating to Medicare reimbursement may have impacted these trends significantly. Between 2009 and 2019, hospital profits for CDA have decreased by as much as −121%, and surgeon reimbursement decreased by about 7%, with only a slight increase in Medicare reimbursement of 15% [50]. Additionally, day-of-surgery reimbursements for revision CDA have been observed to be more than double that of primary CDA, and 90-day global reimbursements were significantly higher for revisions, which may indirectly incentivize more complex revision procedures over primary cases [23]. Changes in clinical guidelines and evolving surgical indications also likely influenced CDA adoption rates. Recent evidence suggests that CDA utilization may have plateaued in part because anterior cervical discectomy and fusion (ACDF) is increasingly indicated in older patients and utilized in outpatient ambulatory surgical centers, and has been seen to possibly provide comparable outcomes, shorter operative times, and favorable economics [[51], [52], [53]]. It is also important to note that despite the growing adoption of CDA, these procedures represent a relatively small proportion of cervical spine surgeries, especially compared to ACDF, which remains a more dominant surgical treatment [21].

Healthcare systems should anticipate potential clinical and economic impacts associated with a large increase in these secondary procedures, including surgical complexity, revision outcomes, and resource allocation. Additionally, the growing trend of removal CDA in the Medicare population may reflect the need to reassess the risk-benefit profile of CDA versus ACDF in older patients. Future studies comparing outcomes between these options in elderly patients are warranted. However, these projections should be interpreted with caution. The observed growth in removal procedures may not apply to younger or commercially insured populations, where risk profile and revision patterns may differ. Additionally, future utilization could be influenced by evolving clinical guidelines, device innovations, or changes in surgical decision-making that are not captured within the scope of our model.

The forecasting methodology applied in this study is consistent with previous spine and orthopedics literature examining procedural trends, and could help inform healthcare planning, surgical decision-making, and policy considerations [1,27,36,54].

Several limitations must be acknowledged. First, projections rely on Medicare Fee-For-Service (FFS) claims data, and upscaling based on Medicare Advantage (MA) proportions introduces uncertainty as procedure ratios may vary based on region. Second, our analysis focuses on Medicare beneficiaries, who are typically 65 years or older, potentially limiting generalizability of these projections to younger patients who may undergo CDA with different growth patterns. Third, as data was derived from procedure volumes and not individual records, information regarding patient level demographics and latest technological techniques were not included. Additionally, patient outcomes such as complication rates, device longevity, or specific indications for removal CDA could not be evaluated. Future studies would benefit from integrating claims data with clinical registries or detailed electronic health record data. Such linkage would help to clarify, for example, whether the observed increase in CDA removals is driven primarily by implant-related conditions, disease progression, patient selection, or variability in surgical technique. The dataset also does not include regional, geographic, or institutional (eg, academic vs. community vs. private practice settings) variation in CDA utilization. CDA adoption and revision rates may vary across regions or institutions due to factors such as local practice preferences, patient demographics, or surgical training access. Therefore, future studies utilizing datasets with detailed geographic or institutional identifiers would be valuable for characterizing local practice patterns and addressing disparities or variability in CDA usage. Revision CDA (CPT 22861) was not analyzed due to CMS data suppression. The scarcity of these procedures may indicate a low occurrence of true revisions relative to removals, or potentially coding practices that possibly categorize procedures as removals rather than revisions. Additionally, our analysis of CDA removals relies on anterior approach CPT codes and may not fully capture revision procedures performed via posterior stabilization or alternative approaches. Thus, the actual number of CDA revisions is likely underestimated. Future studies should analyze granular registry or all-payer claims data to better capture revision CDA procedures and provide insights into its epidemiology. Lastly, while our forecasts are based on observed historical trends, the models do not incorporate unmeasured external factors such as population demographic shifts, regional variation in practice patterns, reimbursement changes, or the introduction of new surgical technologies, all of which could meaningfully alter future CDA utilization.

## Conclusion

Accurately predicting future cervical disc arthroplasty (CDA) procedure volume is challenging due to the evolving surgical technology landscape and shifting patient demographics. However, reliable projections of primary and removal CDA are essential for understanding future healthcare utilization, economic implications, and training requirements. This study builds upon previously validated methods to establish a comprehensive statistical framework for predicting CDA trends in the Medicare population. Primary CDA procedures are anticipated to demonstrate modest growth, while removal CDA may increase more rapidly through 2035, highlighting the need for ongoing surveillance and strategic resource planning.

## Funding

No internal or external funding was utilized for this study.

## Declaration of competing interest

No conflicts of interest.
